# Establishment of a clinical network for children with amelogenesis imperfecta and dentinogenesis imperfecta in the UK: 4-year experience

**DOI:** 10.1007/s40368-023-00859-2

**Published:** 2024-02-03

**Authors:** J. Monteiro, R. Balmer, F. Lafferty, A. Lyne, A. Mighell, K. O’Donnell, S. Parekh

**Affiliations:** 1 Sheffield Teaching Hospitals, University of Sheffield, Sheffield, UK; 2 School of Dentistry, The University of Leeds, Leeds, UK; 3 Glasgow Dental Hospital, Glasgow, UK; 4 RNENTEDH, University College London Hospitals, London, UK; 5 Leeds Dental Hospital, Leeds, UK; 6 UCL Eastman Dental Institute, London, UK

**Keywords:** Amelogenesis imperfecta, Dentinogenesis imperfecta, Clinical excellence group, Genetic testing, Peer support

## Abstract

**Background:**

*Amelogenesis imperfecta (AI)* and *dentinogenesis imperfecta* (DI) are two groups of genetically inherited conditions resulting in abnormal enamel and dentin formation, respectively. Children and young people may be adversely affected by these conditions, with significant reduction in oral health related quality of life. Dental management of children with AI and DI is often complex, which is exacerbated by the absence of clear referral pathways and scarce evidence-based guidelines.

**Method:**

The need for increased knowledge and peer support led to the development of a group of UK paediatric dentists with a special clinical interest in the management of children with AI and DI.

**Purpose:**

The aims of this paper are to describe the establishment of an AI/DI Clinical Excellence Network (AI/DI CEN) in paediatric dentistry including outputs and future plans, and to share our collective learning to help support others anywhere in the world advance the care of people with AI or DI.

## Background

**Fig. 1:**
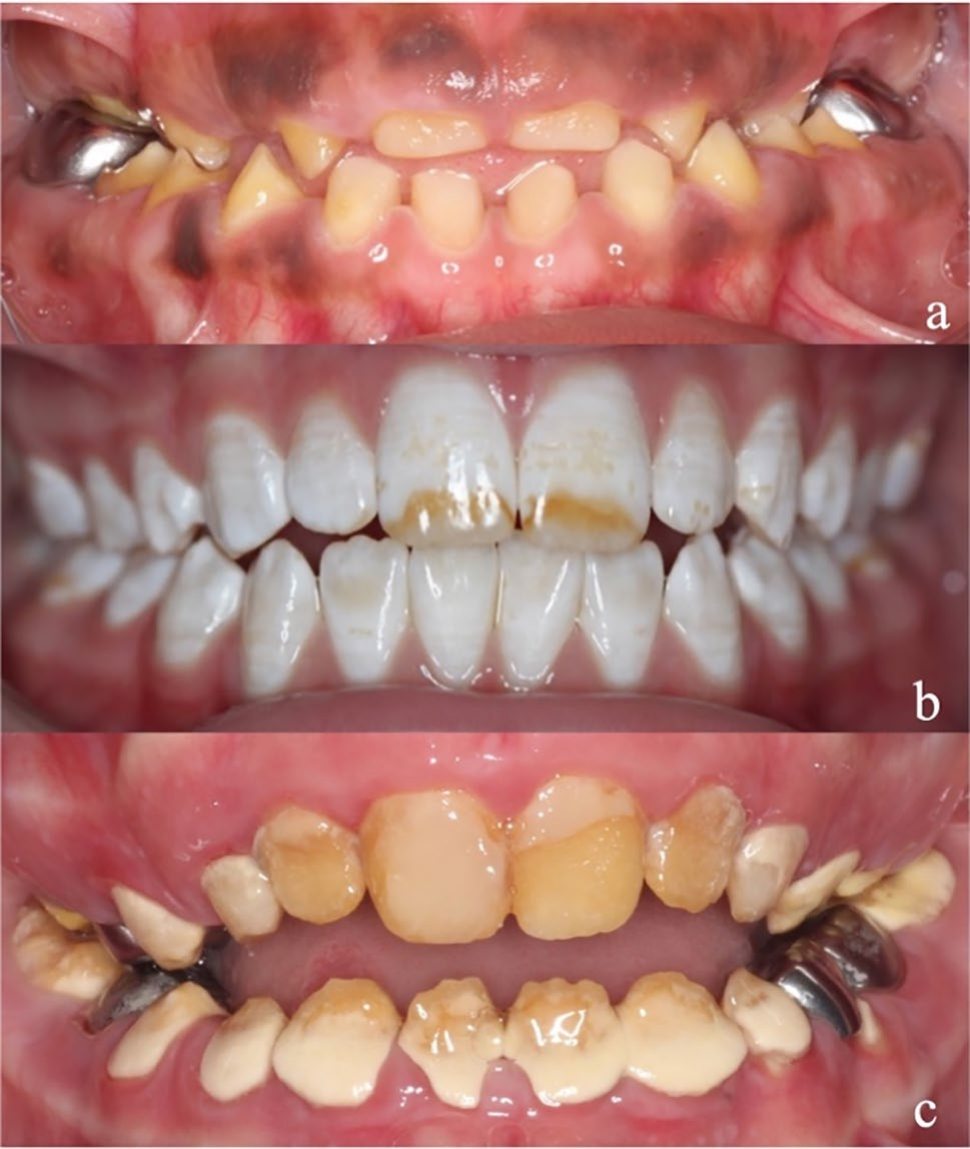
Examples of the three major AI types. **a** Anterior photograph of a child with hypoplastic AI in the permanent dentition; **b** anterior photograph of a child with hypomaturation AI in the permanent dentition; **c** anterior photograph of a child with hypocalcified AI showing calculus, enamel breakdown and restoration failure due to decreased composite bond strength

*Amelogenesis imperfecta (AI)* is a heterogeneous group of genetically inherited conditions resulting in abnormal enamel formation in all teeth of both dentitions. It is a rare disorder with poorly understood prevalence, likely due to population variations and different study methodologies. The Witkop classification has developed over time with major types of AI described by clinical features including hypoplastic, hypomaturation, hypocalcified AI and hypomaturation–hypoplastic with taurodontism, with recognition of links to developmental dentine disorders (1988) (Fig. 1). Witkop restricted the term AI to situations where this occurred in isolation of other, co-segregating health issues. As genetic studies have brought new insight into the molecular basis to inherited enamel defects, there has been greater recognition of how AI links to wider health issues, including to some named syndromes (Smith et al. 2017; Wright 2023). It also offers new opportunities in improving understanding the boundaries between what is AI and what are enamel defects due to other reasons (Collignon et al. 2022). This fast evolving knowledge offers greater insight into AI, with the potential of genetic diagnosis and how this impacts the management of different AI types yet to be realised (Smith et al. 2017; Wright 2023).

Children, young people and their families can be adversely affected by AI, with significant reduction in oral health-related quality of life (OHRQoL). Most issues relate to aesthetic and hypersensitivity concerns, with children reporting bullying and difficulties eating due to pain (Lyne et al. 2021). Furthermore, children find dental treatment difficult, often feeling that dentists do not understand the condition or address their concerns (Parekh et al. 2014; Pousette Lundgren, Karsten and Dahllöf, 2015a; Pousette Lundgren et al. 2015a, b; Pousette Lundgren et al. 2016). This poses considerable issues, as treatment of AI is protracted, particularly until all the teeth have erupted, with a considerable commitment to ongoing care by the children and their families (Lafferty et al. 2021).

**Fig. 2:**
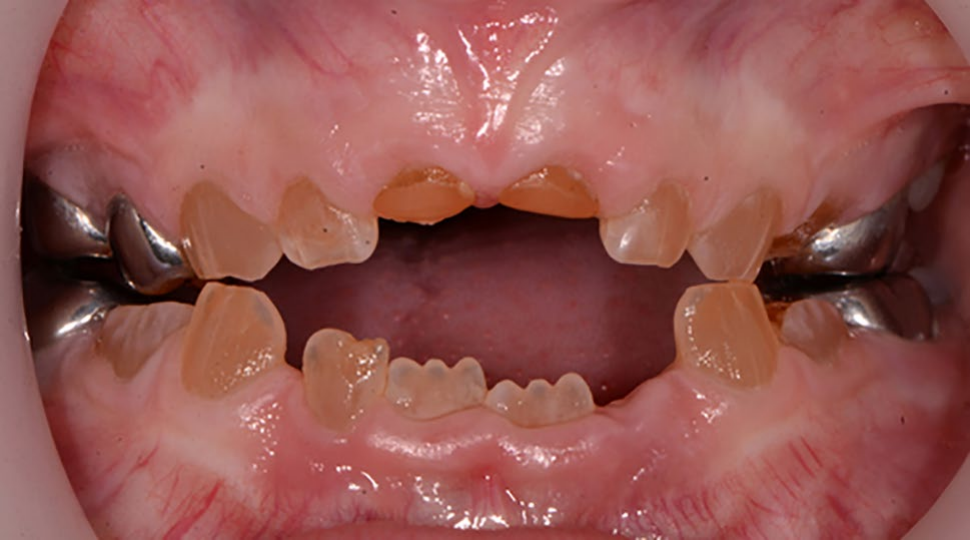
Anterior photograph of a child with dentinogenesis imperfecta (DI)

*Dentinogenesis imperfecta (DI)* and dentine dysplasia are autosomal dominant disorders, where dentine forms abnormally (de La Dure-Molla, Philippe Fournier and Berdal, 2015). Patients present with discoloured, opalescent dentine that can wear easily, with frequent enamel breakdown. Patients have reported aesthetic and functional limitation; however, due to pulp canal obliteration, they rarely have pain or dental infection (Fig. 2). This condition in some specific instances is associated with osteogenesis imperfecta (OI). Increasing use of bisphosphonates in OI treatment should be considered when treatment planning for these cases. Although there are no known reports of medication-related osteonecrosis of the jaws (MRONJ) in children, care should be given to adequate medium- and long-term planning and prevention is paramount (Neal and Schlieve 2022).

From a clinician’s perspective, AI and DI present multiple challenges including clear pathways for the patients to reach specialist services in a timely way, continued attendance over many years and appropriate transfer of children to adult services. Furthermore, there are often difficulties with bonding, increased dental anxiety, associated dental anomalies and/or orthodontic malocclusion. These are rare conditions, so the literature on evidence-based care is scarce and mostly reliant on case reports, with rare randomised control trials and systematic reviews (Dashash et al. 2013). The need for increased knowledge and peer support led to the development of a group of UK paediatric dentists with a special clinical interest in the management of children with AI and DI. The need for a more coordinated approach to patient care within a supportive environment is clear.

The aims of this paper are to describe the establishment of an AI/DI clinical excellence network (AI/DI CEN) in paediatric dentistry including outputs and future plans, and to share our collective learning to help support others anywhere in the world advance the care of people with AI or DI.

## Setting up of the AI/DI CEN

The plan for an AI/DI CEN started to take shape in 2018, following an informal meeting of senior specialists in paediatric dentistry and oral medicine with special interest in AI. Having identified common challenges, it was felt that a collaborative approach to research, quality improvement, teaching/peer support and dissemination to others would ultimately lead to improved patient care. In March 2019 a half-day AI workshop for the British Society of Paediatric Dentistry’s (BSPD) consultant group (CGPD) was held in Leeds. With overwhelming engagement, several working groups were created to address different issues related to managing children with AI and DI. The AI/DI CEN was formally created and currently actively engages with over 60 paediatric dentists and trainees from the UK and Ireland.

The COVID-19 pandemic presented a challenge, as well as an opportunity. Like in so many areas, several projects were interrupted or cut short by the pandemic and planned face-to-face training had to be organised online. This provided an excellent opportunity to eliminate financial and geographic constraints, making training accessible to a greater number of specialists/trainees.

## Organisational structure

**Fig. 3:**
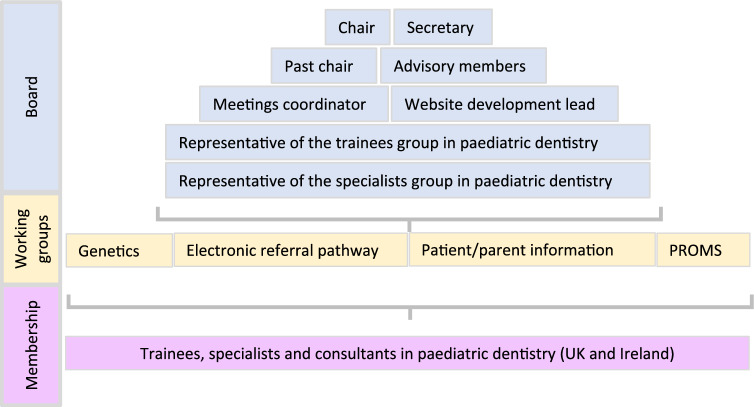
Organisational structure of the AI/DI CEN

A working committee was established to formalise the CEN. Board members were agreed upon, including chair, secretary and meeting coordinator (Fig. 3). Terms of reference were created, addressing representation, roles, tenure of appointments, dissemination and accountability. The terms of reference were circulated and agreed by those engaged with the CEN.

The AI/DI CEN’s aims to strengthen management and collaboration between clinical teams nationally in the care of patients with AI/DI (Table 1). The AI/DI CEN’s objectives are to:

1. Provide an inclusive forum for health-care professionals to share, discuss and improve the diagnosis, treatment provision and management of children with AI and DI with successful transition to adult care services.
2. Develop national dentist-led genetic testing for children with AI.
3. Advise national groups and relevant bodies on the dental care of children with AI and DI.
4. Develop and disseminate evidence-based practice linked to standards on the dental management and care pathways of children with AI and DI.
5. Undertake collective national quality improvement and research to improve the oral health and care pathways of children with AI and DI and make best use of NHS funding.
6. To support professional development of educational resources for dental professionals involved in the management of children with AI and DI.
7. To develop educational resources for patients and families with AI and DI in partnership with the users.
8. To involve children and families in development of resources, pathways, quality improvement projects and research.

**Table 1:** AI/DI CEN’s collaborative publications and ongoing/planned projects

AI/DI CEN publications				
Author/year	Description	Number of units	Number of participants	Main outcomes
Lyne et al. (2021)	Multi-centre service evaluation of specialist paediatric dentistry services in the UK to evaluate patient-reported outcome measures (PROMS) for children with AI	6	60 children	• 72% 'often' or 'sometimes' experienced pain or sensitivity • 76% 'often' or 'sometimes' felt unhappy with the way their teeth look • Happy with their teeth: 81% post-treatment, 41% mid-treatment and 33% pre-treatment
Lafferty et al. (2021)	Multi-centre, retrospective analysis of the burden of care in AI paediatric dentists (UK)	3	138 children	• Average patient age at first referral: 7.7 years (range 1–16 years) • Families travelled an average 21.8 miles per appointment (range 0.2–286 miles) • Patients attended on average 4.5 appointments per year for 5.8 years • In total, 65.2% had experience of local anaesthetic, 27.5% inhalation sedation and 31.9% general anaesthetic • Dental treatment including restorations and extractions was commonly required on multiple teeth per patient
Wood et al. (2022)	PROMS of children undergoing dental whitening (including AI, DI, molar incisor hypomineralisation and trauma)	3	27 children	• Patients reported improvements in their appearance (89%) and self-confidence (81%) • Sensitivity was the most common side effect (63%)
Lafferty et al. (2021)	Online survey of BSPD members to assess the provision of specialist care and transitional care arrangements for paediatric patients with AI	N/A	66 dentists	• 49% (*n* = 47) reported having no clear AI referral pathway for specialist care • 77% (*n* = 72) reported having no transitional care pathway with 85.9% (*n* = 73) indicating the need for one

• AI/DI CEN’s ongoing and planned projects		
Project description	Start date	Status
Electronic pathway in Yorkshire, aiming to improve AI care, guide MDT management and access to genetic testing		Ongoing
AI PROMS (longitudinal, ongoing collection)	2021	Ongoing
Development of an AI diagnostic aid resource	2021	Undergoing consultation by AI/DI CEN,
Development of AI treatment flowcharts	2022	Undergoing consultation with AI/DI CEN
Development of a website with information about AI/DI for affected patients and the dental team involved with their care	2021	Ongoing
Patient resources development	2021	Ongoing
Hypomineralisation/hypoplasia calibration tool	2023	Ongoing
Dentists-led genetic testing for isolated DI	2023	Ongoing
DI PROMS	2023	Ongoing

## Quality improvement projects and research

Several collaborative projects were developed, including service evaluations and research resulting in publications (Table 1). Ongoing and planned projects include the development of electronic care pathways for children with AI, continuation of the AI PROMS, development of an AI diagnostic aid and of AI treatment flowcharts (Table 1).

## Education

At the time of writing, the AI/DI CEN has organised eight biannual workshops: an online half-day workshop in the winter, and a study day in the spring (initially online, now in hybrid format). These sessions cover different aspects of AI and DI, including AI genetic testing (joint training with Genomics England), AI/DI classification, phenotype, treatment and patient/carers involvement. They aim to provide widespread training and support to clinicians, including trainees and specialists/consultants in paediatric dentistry, and have received excellent feedback. In line with this ethos, organisers and speakers work on a voluntary basis and training is free of charge, with support from Health Education England Yorkshire and Humber (HEEYH) and the universities of Leeds, Sheffield and University College London. Talks are run by experts in AI/DI, including CEN board members and with invited national and international speakers. Trainees are routinely invited to present their AI/DI projects and cases. An important feature of these training sessions has been enabling discussion between facilitators and attendees, which has received excellent feedback and provides peer support. Each workshop has had about 70 attendees, which is just below a quarter of the specialist/consultant/trainee workforce in the UK. This undoubtedly proves the need for training and peer support.

## Peer engagement

Communication is made through subscribed mailing lists, including an annual bulletin, describing the work of the CEN throughout the year. More recently, the AI/DI CEN team has been invited to provide teaching on a pre-congress workshop of the International Association of Paediatric Dentistry (IAPD) conference in Maastricht in June 2023 that drew on the format used AI/DI CEN Meetings.

## Collaboration with Genomics England to support uptake of genetic testing for AI

AI genetic testing as part of National Health Service (NHS England) care is available in England via the National Genomic Directory and includes a panel of 40 known affected genes (NHS England 2021). Testing is requested by specialist dentists with expertise in developmental dental defects or clinical genetics services and is under the umbrella of the directory for rare and inherited diseases, commissioned by the NHS in England. McDowall and colleagues (2018) identified knowledge and attitudes of paediatric dentists in relation to genetic testing for AI. The authors identified the need to upskill the specialty, specifically regarding training for indications, requesting, consenting, results interpretation and discussion of with families. With this in mind, the AI/DI CEN developed workshops for indications, consent and discussion of genetic test results, in collaboration with Genomics England education team. Collaborative development of resources, including standard operating procedure, information sheets, consent forms and patient letters have supported different units in introducing this resource. The uptake of NHS AI genetic testing has increased with a number of specialist paediatric dentistry units in England providing this service to their patients as part of regular care.

## Future plans

The AI/DI CEN vision include continuing to develop training sessions and peer support, as well as developing further research and service improvement collaborations. Development of online resources is a priority, to include the ongoing work with Genomics England and patient resources. Alongside peer support, the AI/DI CEN are working to produce patient resources and keen to promote patient/carer involvement. Representation of different groups in the board has been identified as one of the priorities for this year, along with collaboration with Restorative Dentistry groups, brought by requests for training from trainees in this specialty.

## How to set up a regional AI CEN group?

The establishment of a regional AI/DI CEN group is highly encouraged, given our positive experiences and clear appetite to sustain the approach over time. We recommend assessing which regional resources are already in place and discussing with clinicians who manage children and young people with AI, to identify challenges and training needs of the group. Communication with patients and their families is crucial, as high quality patient care and support must always be the utmost priority. Finally we suggest, engaging the wider dental and other healthcare communities and providing a point of support and training.

**Fig. 4:**
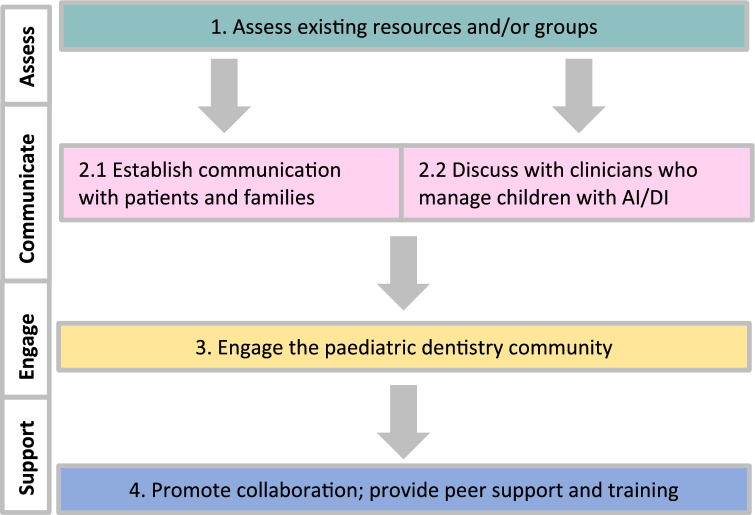
Flowchart of recommendations for establishment on an AI/DI CEN

At a European/international level, the group has established working collaborations with different world experts, with participation in an IAPD course. Support from EAPD would be valuable in establishing a European network. Figure 4 provides a flowchart with suggested flowchart for the establishment of an AI/DI CEN (Fig. 4).

## Conclusion

In the 4 years since its formation, the AI CEN has developed from a gathering of interested individuals to a highly engaged and dynamic group, with a number of ongoing collaborations and publications, whilst providing support to peers and developing the next generation of British paediatric dentists. Through challenges and opportunities the AI/DI CEN is willing to continue working to support colleagues and improving care to such a challenging as well as rewarding paediatric patient group.
